# The synthetic peptide P111-136 derived from the C-terminal domain of heparin affin regulatory peptide inhibits tumour growth of prostate cancer PC-3 cells

**DOI:** 10.1186/1471-2407-11-212

**Published:** 2011-05-30

**Authors:** Yamina Hamma-Kourbali, Oya Bermek, Isabelle Bernard-Pierrot, Racha Karaky, Dominique Martel-Renoir, Sophie Frechault, José Courty, Jean Delbé

**Affiliations:** 1 Laboratoire de Recherche sur la Croissance Cellulaire, la Réparation et la Régénération Tissulaires (CRRET), Université Paris Est Créteil, CNRS, avenue du Général de Gaulle, 94010 Créteil Cedex, France; 2 CNRS, Vectorologie et Transfert de Gènes, Institut Gustave Roussy PR2, 38 rue Camille Desmoulins 94805 Villejuif Cedex, France; 3 The present address of I. Bernard-Pierrot is UMR 144, CNRS-Institut Curie, 26 rue d'Ulm, 75248 Paris Cedex 05, France

## Abstract

**Background:**

Heparin affin regulatory peptide (HARP), also called pleiotrophin, is a heparin-binding, secreted factor that is overexpressed in several tumours and associated to tumour growth, angiogenesis and metastasis. The C-terminus part of HARP composed of amino acids 111 to 136 is particularly involved in its biological activities and we previously established that a synthetic peptide composed of the same amino acids (P111-136) was capable of inhibiting the biological activities of HARP. Here we evaluate the ability of P111-136 to inhibit *in vitro *and *in vivo *the growth of a human tumour cell line PC-3 which possess an HARP autocrine loop.

**Methods:**

A total lysate of PC-3 cells was incubated with biotinylated P111-136 and pulled down for the presence of the HARP receptors in Western blot. *In vitro*, the P111-136 effect on HARP autocrine loop in PC-3 cells was determined by colony formation in soft agar. *In vivo*, PC-3 cells were inoculated in the flank of athymic nude mice. Animals were treated with P111-136 (5 mg/kg/day) for 25 days. Tumour volume was evaluated during the treatment. After the animal sacrifice, the tumour apoptosis and associated angiogenesis were evaluated by immunohistochemistry. *In vivo *anti-angiogenic effect was confirmed using a mouse Matrigel™ plug assay.

**Results:**

Using pull down experiments, we identified the HARP receptors RPTPβ/ζ, ALK and nucleolin as P111-136 binding proteins. *In vitro*, P111-136 inhibits dose-dependently PC-3 cell colony formation. Treatment with P111-136 inhibits significantly the PC-3 tumour growth in the xenograft model as well as tumour angiogenesis. The angiostatic effect of P111-136 on HARP was also confirmed using an *in vivo *Matrigel™ plug assay in mice

**Conclusions:**

Our results demonstrate that P111-136 strongly inhibits the mitogenic effect of HARP on *in vitro *and *in vivo *growth of PC-3 cells. This inhibition could be linked to a direct or indirect binding of this peptide to the HARP receptors (ALK, RPTPβ/ζ, nucleolin). *In vivo*, the P111-136 treatment significantly inhibits both the PC-3 tumour growth and the associated angiogenesis. Thus, P111-136 may be considered as an interesting pharmacological tool to interfere with tumour growth that has now to be evaluated in other cancer types.

## Background

Prostate cancer is among the leading malignancies in men throughout much of the industrialized world and ranks second among causes of death from cancer. The lack of not enough effective treatments indicates a need to develop novel treatment strategies targeting new molecules like growth factors. Epithelial-stromal interactions play a pivotal role in the functional integrity of the normal prostate adult gland [1]. This physiological process requires complex interactions between peptide growth factors and growth modulators, which may be regulated either by androgens or by other factors [2,3]. Any imbalance in these interactions, such as up or down regulation of growth factors or their receptors or a switch from paracrine to autocrine mediation of growth-factor pathways leads to prostate tumour progression. Among the growth-factor families involved in prostate-cancer progression, Transforming Growth Factor-beta (TGFβ), Fibroblast Growth Factors (FGFs), Epidermal Growth Factor (EGF) and heparin affin regulatory peptide (HARP) were reported to play a prominent role [3]. HARP, also called pleiotrophin is a 136 amino acids secreted polypeptide that forms with the protein midkine (MK) a specific family among the heparin-binding growth factors [4]. During embryonic development, HARP is expressed in tissues originating in the mesoderm and neuroectoderm, suggesting a role in epithelium-mesenchyme interactions and in neuronal migration. In adults, HARP expression is limited except at sites such as the mammary gland and uterus associated with reproductive angiogenesis [2]. Furthermore, HARP overexpression has been documented in pathologies associated with cell proliferation and angiogenesis, such as rheumatoid arthritis [5] and tumour growth [6]. HARP has been shown to exert oncogenic potential by transforming various cell lines upon HARP cDNA transfection [7,8]. In addition, HARP has been shown to play a key role in prostate cancer. Thus, plasma HARP levels were elevated in patients with prostate cancer [9,10]. Furthermore, HARP protein was associated with epithelial cells in prostate cancer but not in normal prostate tissue and the mRNAs were located in the stromal compartment, suggesting a paracrine mechanism of action for HARP [11]. *In vitro*, HARP overexpression in normal prostate epithelial PNT-1A cells induced both anchorage-independent and anchorage-dependent growth at low serum concentrations. HARP was also mitogenic for PC-3, LNCaP, and DU145 cell lines [11]. The growth-promoting effect of HARP on prostate cancer cells was also confirmed using an antisense strategy, which established HARP as an important autocrine growth factor for the LNCaP prostate-cancer cell line and as a paracrine factor involved in angiogenesis [12].

Two transmembrane proteins with intracellular catalytic domains have been described as HARP receptors: the receptor-type protein tyrosine phosphatase beta/zeta (RPTP β/ζ) and the anaplastic lymphoma kinase (ALK) receptor. The mitogenic and anti-apoptotic activities of HARP were initially linked to the high-affinity tyrosine kinase receptor ALK in a process mediated by the phosphatidylinositol 3-kinase and MAP kinase signaling pathways [13]. ALK was first identified as a constitutively active, oncogenic, chimeric nucleophosmin-ALK fusion protein [14]. Like HARP, ALK is expressed during normal embryonic development in the similar pattern [15]; however it is also overexpressed in different several human cancers [16,17]. The neurite outgrowth, the cell migration and adhesion activities of HARP were initially associated with the chondroitin sulfate proteoglycan RPTP β/ζ [18,19]. HARP was shown to signal through enforced dimerization of RPTPβ/ζ which, in turn, results in a loss of the RPTPβ/ζ catalytic tyrosine phosphatase activity. Inactivation of RPTPβ/ζ by HARP interaction increased tyrosine phosphorylation of each of the substrates of RPTPβ/ζ leading to modification of the cytoskeleton network via the β-catenin, p190RhoGAP, and β-adducin signaling pathway [20-23]. However, it has also been recently demonstrated that ALK is activated through the HARP/RPTP β/ζ pathway, thus introducing a unique "alternative mechanism of tyrosine kinase receptor activation" [24].

HARP consists of two beta-sheet domains (N- and C-TSR) containing thrombospondin-I repeats, which are flanked by flexible lysine-rich N- and C-terminal tails. Previous studies suggested that the C-TSR domain and the C-terminus regions of HARP were particularly involved in the biological activities of HARP [25-28]. Thus, the C-terminus, composed of amino acids 111 through 136, was involved in the binding of HARP to the ALK receptor [25,26]. In these previous studies, mutant HARP protein lacking amino acids 111 through 136 acted as a dominant negative effector of HARP mitogenic, angiogenic, and transforming activities. The synthetic peptide composed of the deleted amino-acid segment P111-136 inhibited HARP mitogenic activity in NIH-3T3, the growth of MDA-MB 231 cells in soft agar and competed with HARP for binding to the extracellular domain of ALK receptor [26] thus representing an interesting pharmacological tool.

The objective of this study was to further complete our investigations on P111-136 and to evaluate its growth inhibitory activity both *in vitro *and *in vivo *on the human prostatic adenocarcinoma cell line PC-3.

## Methods

### Materials

Culture mediums, foetal calf serum (FCS), G418 and rabbit antibody to human ALK were supplied by Invitrogen (Cergy Pontoise, France). Heparin-Sepharose, streptavidin-Sepharose gels and Mono-S™ column were from GE HealthCare (Orsay, France), BM ChemiLuminescence from Roche Diagnostic (Meylan, France). Superblocker™ solution was purchased from Perbio-Pierce (Montluçon, France). Immobilon-P from Millipore Corp (Saint-Quentin en Yvelines, France). Goat antibody to human HARP and rabbit antibody to human RPTPβ/ζ were from R&D (Lille, France). Monoclonal antibody to human nucleolin was from Santa Cruz biotechnology (Montrouge, France), goat anti-mouse CD31 (PECAM) polyclonal antibody and Matrigel™ were from BD Pharmingen Biosciences (le Pont de Claix, France). Horseradish peroxidase-conjugated rabbit anti-goat IgG and goat anti-rabbit IgG were purchased from Jackson (Montluçon, France). Taxol^® ^was purshased from Sigma Aldrich (Saint Quentin Fallavier, France). Synthetic P111-136 (KLTKPKPQAESKKKKKEGKKQEKMLD) and biotinylated P111-136 (KLTKPKPQAESKKKKKEGKKQEKMLDK-biot-CONH2) were obtained from Altergen (Schiltigheim, France). Recombinant human HARP was produced and purified from mammalian culture as described previously [25].

### Colony formation in soft agar

PC-3 cells (ATCC, Manassas, VA, USA) were seeded at a density of 2 × 10^4 ^in triplicate into 12-well plates containing agar and DMEM supplemented with 5% FCS and various concentrations of either P111-136 peptide or anti-human HARP antibody or control non immune immunoglobulins. The compounds were added to the culture medium twice a week. After 12 days, colonies larger than 50 μm in diameter were counted using a phase-contrast microscope equipped with a reticule, in five fields in each of three wells. The assay was repeated at least twice.

### Pull down and Western blot analysis

Cells were cultured in 100 mm Petri dishes in complete medium, then media were discarded and cells were washed 3 times with ice-cold PBS and lysed with 1 ml of lysis buffer (50 mM HEPES, pH 7, 150 mM NaCl, 10 mM EDTA, 1% TritonX-100, 1% Nonidet P-40 (both v/v), 1 mM phenylmethylsulfonyl fluoride, 1 mM sodium orthovanadate, 5 μg/ml aprotinin, and 5 μg/ml leupeptin). For pull down experiments, total proteins from cell lysate were precleaned with streptavidin-Sepharose beads for 1 h at room temperature followed by centrifugation at 10,000 × g for 5 min. The beads were collected by centrifugation and the supernatants were transferred to new microfuge tubes. After this precleaning step, supernatants were incubated overnight at 4°C with 5 μM of biotinylated P111-136. A suspension of streptavidin-agarose beads in a volume of 80 μl was added. After 3 h incubation at 4 °C, beads and bound proteins were collected by centrifugation (10,000 × g, 4°C) and washed by centrifugation three times with ice-cold cell lysis buffer. The pellet was resuspended in 60 μl of 2 × SDS loading buffer (100 mM Tris-HCl, pH 6.8, 4% sodium dodecyl sulfate (w/v), 0.2% (w/v) bromophenol blue, 20% glycerol, 0.1 M dithiothreitol), and kept at 4 °C until use. Western blot experiments were performed as described previously [29] using either anti RPTPβ/ζ, anti ALK or anti nucleolin antibodies. For the presence of HARP in PC-3 conditioned media, experiments were performed as described in previous studies [25].

### Tumour-cell inoculation to nude mice

All *in vivo *experiments were approved by the appropriate ethics committee and conducted in compliance with European Community directives. PC-3 carcinoma cells (2 × 10^6^) were injected subcutaneously in the right flank of female athymic nude mice (Janvier, Le Genest St Isle, France). Two weeks after the injection, mouse had one palpable tumour about 60 mm^3 ^in size. Groups of 5 mice were then given peritumoral injections of either 0.1 ml PBS alone, Taxol^® ^10 mg/kg twice a week, or P111-136 (5 mg/kg/day). Tumour size was determined twice a week by using callipers to measure the lengths of the two main axes, computing the corresponding radii (labelled R1 and R2, with R1 < R2), and estimating tumour volume as V = 4/3 × π × R_1_^2^xR_2_, where R_1 _is radius 1, R_2 _is radius 2 and R_1 _< R_2_.

### Tissue preparation, immunohistochemical staining, and image analysis

The PC-3 tumours were removed surgically then immediately quick-frozen in liquid nitrogen and fixed for 20 min in acetone at 4°C. The 6-μm sections were rehydrated in PBS then saturated with PBS containing 1% bovine serum albumin (BSA) and 2% normal goat serum. Endogenous biotin was blocked using the Vector blocking kit (Vector Laboratories, Burlingame, CA). To visualize endothelial cells within the tumours, sections were incubated with goat anti-mouse CD31 polyclonal antibody for 1 h at room temperature. After two washes in PBS-Tween 20 (0.2% v/v), sections were incubated for 1 h at room temperature with biotinylated goat anti-rabbit IgG (Chemicon International Inc., Temecula, CA) in saturation buffer, followed by three washes and incubation with an avidin-biotinylated-alkaline phosphatase complex (Vector Laboratories). Alkaline phosphatase activity was revealed using the Vector red substrate (Vector Laboratories). Finally, the sections were counterstained with haematoxylin, washed with water, and cover slipped with mounting medium (Thermo Shandon, Pittsburgh, PA). For each CD31-labelled section, five microscopic fields containing exclusively viable tumour cells were randomly selected for analysis. Endothelial-cell density was expressed as the ratio of endothelial-cell area/total area examined × 100. Mean values were then computed for untreated and treated tumours.

For tumour apoptosis analysis, immunohistochemistry was performed as previously described [30] using rabbit antibody raised against cleaved caspase 3 (Cell Signaling Technology, Saint-Quentin en Yvelines, France). Quantification of staining on histological slides was achieved using PIXCYT; a software package [31] designed by the Groupe Régional d'Etudes sur le Cancer, (Centre François Baclesse, Caen, France). This system combines a dedicated slide scanner and a computer-assisted image analysis.

### *In vivo *mouse angiogenesis assay using the Matrigel™ plug model

Swiss mice (Janvier, Le Genest St Isle, France) were injected subcutaneously with 0.3 ml of growth-factor reduced Matrigel™ alone or containing either P111-136 (1 μM), HARP (5 nM), or a mixture of HARP and P111-136, (4 mice/group). The Matrigel™ rapidly formed a single, solid, gel plug. After 8 days, the skin was pulled back to expose the intact plug, which was dissected out, frozen in liquid nitrogen, and fixed with acetone. Matrigel™ plug sections 8 μm in thickness were cut using a cryostat CM3050 (Leica Microsystems, Rueil, France) and stained with Gomori-Trichrome for microscopic observation. The area infiltrated by endothelial cells was then measured using an image analyser in six fields in each of three Matrigel™ plug sections from each mouse.

### Statistical analysis

Unpaired Student's t test was used to assess differences between each group and the corresponding control group. Each experiment includes triplicate measurements for each condition. All results are reported as mean ± SD determined from at least two independent experiments.

## Results

Previously, it has been shown that peptide P111-136, structurally related to the C-terminal domain of HARP, inhibited specifically the mitogenic activity induced by HARP. It was also shown that P111-136 inhibited the *in vitro *growth of the human breast cancer cell line MDA-MB 231 suggesting that P111-136 blocked the autocrine stimulation loop of HARP. ***I***n order to investigate more precisely the effect of P111-136 on tumour growth, including *in vivo *study, we investigated the effect of P111-136 on the androgen-independent prostate-cancer cell line PC-3 in which the presence of an autocrine loop of stimulation for this growth factor has been suggested [11,32].

### The peptide P111-136 inhibits the growth of PC-3 cells

**Figure 1:**
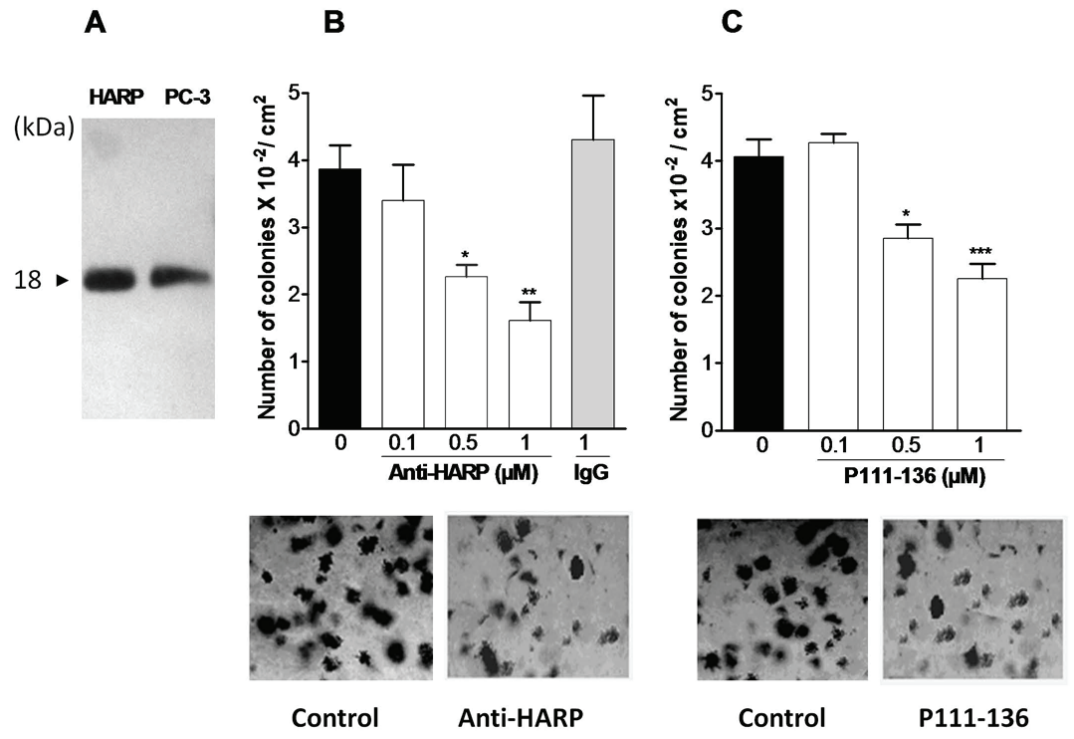
**Inhibition of PC-3 colony formation by peptide P111-136 and association with the HARP receptors**. (A), Western blot analysis of HARP in the conditioned media of PC-3 cells (PC-3), 20 ng of recombinant HARP was used as reference. (B), polyclonal anti-human HARP antibody or control IgG or (C), P111-136 peptide were added to the culture medium twice a week. After 12 days, colonies with diameters greater than 50 μm were identified using a phase-contrast microscope equipped with a reticule. Representative pictures of colonies are shown below each treatment. Scale bar, 100 μm. Asterisks denote statistically significant differences with the corresponding untreated cells. Data are the means +/- SD of two experiments, each carried out in triplicate. *0.01 <*p *< 0.1, **0.001 <*p *< 0.01, and *** *p *< 0.001.

To confirm the HARP autocrine loop in supporting the growth of PC-3 cells, the presence of HARP in the conditioned media of PC-3 was firstly investigated by Western-blot. As shown in Figure 1A, a weak immunoreactive signal was observed in accord with previous observations [32]. Secondly, to prove that secreted HARP acted on PC-3 cells, the growth of these cells was studied in the soft agar colony-formation assay, a hallmark of malignant transformation, in absence or presence of a polyclonal anti-HARP antibody. As presented in Figure 1B, the polyclonal anti-HARP antibody induced a dose-dependent decrease in colony formation, whereas no effect has been observed using the idiotypic immunoglobulins as the control. These data confirmed the existence of an autocrine HARP-signalling loop for PC-3 cells and prompted us to investigate the effect of P111-136 on the proliferation of this cell line. P111-136 dose-dependently decreased colony formation (Figure 1C). In the presence of 1 μM P111-136, the colonies were smaller and 47% less numerous, compared to the control performed without peptide. In this respect, it is noteworthy that when tested in the same conditions, P122-131, which is another peptide derived from the C-terminal part of HARP inhibiting the growth of DU145 and LNCap prostate cancer cells [33], acted also on PC-3 cells but in a lesser extent with only 22% inhibition for 1 μM (data not shown).

### The peptide 111-136 inhibits the tumour growth of PC-3 cells in a mice xenograft model and the associated angiogenesis

**Figure 2:**
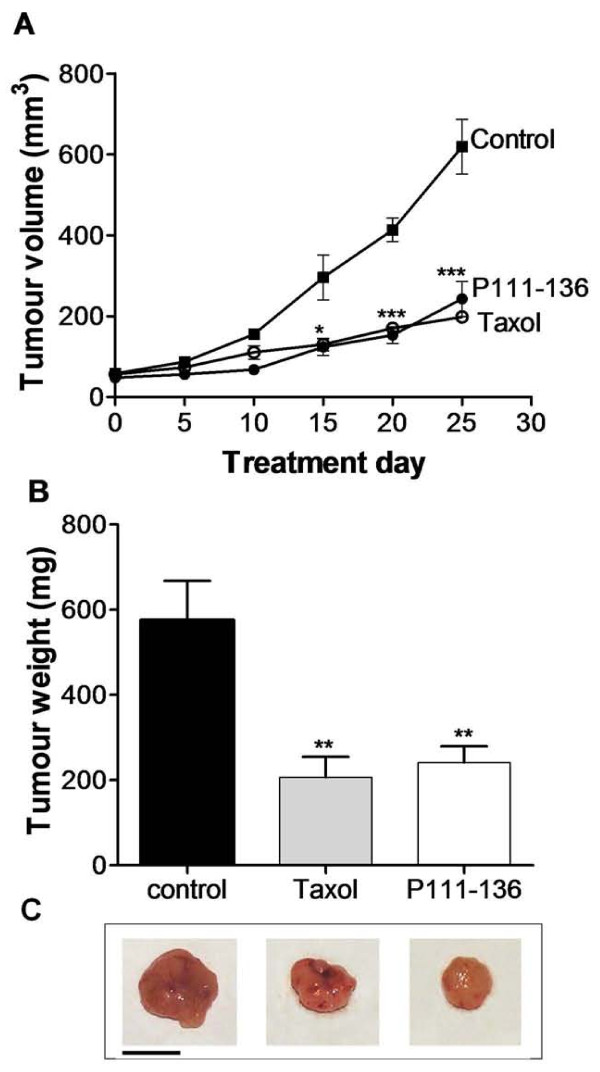
**Inhibition of tumour growth by peptide P111-136**. (A), Tumour-growth curves. PC-3 carcinoma cells (2 × 10^6^) were injected subcutaneously into the right flanks of female nude mice. When tumour size was about 60 mm^3^, mice received peritumoral injections of P111-136 (5 mg/kg/day) (black circle), Taxol^® ^(10 mg/kg twice a week) (white circle), or PBS used as vehicle. (black square) for 25 days. Tumours were measured twice a week. (B), The mice were sacrificed 25 days after the cell injection; the tumours were excised and weighed. (C), A representative excised tumour of each group was represented below. Scale bar, 1 cm. The data are mean tumour volume or weight +/- SD obtained from 5 mice in each group. *p < 0.05, **p < 0.01, and ***p < 0.001 versus control (untreated tumours).

**Figure 3:**
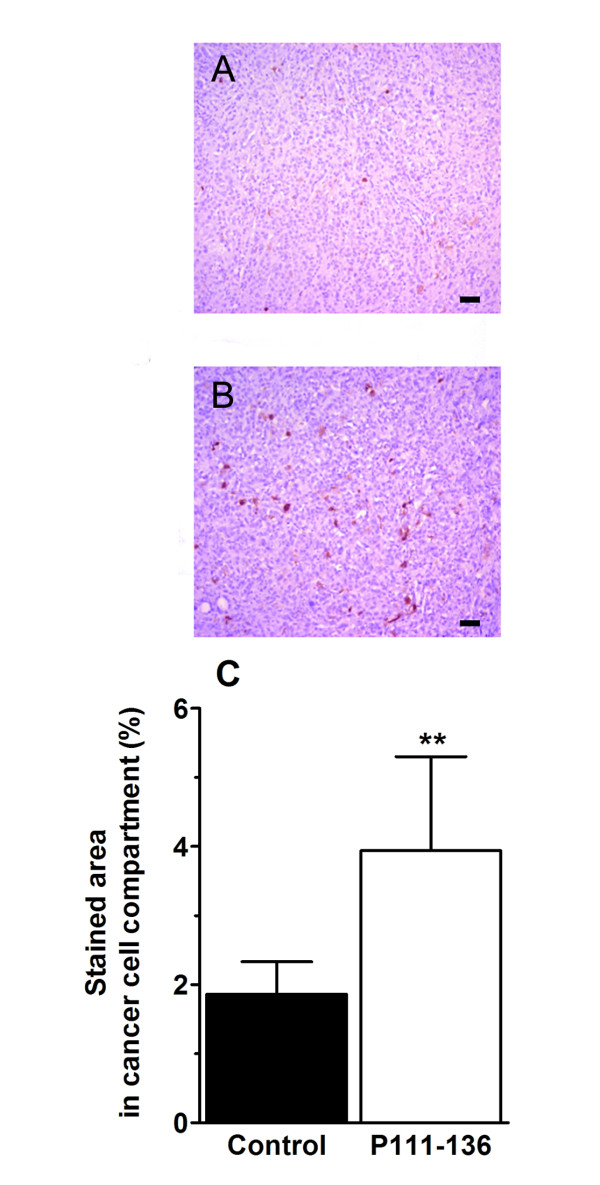
**Induction of apoptosis in xenograft tumour by P111-136**. PC-3 tumour apoptosis was evaluated on tumour sections using immunohistochemistry with antibody directed against cleaved caspase 3. (A), untreated tumours and (B), tumours treated with P111-136. Scale bar, 100 μm. (C), apoptosis quantified by image analysis of caspase 3-labelled cells on the whole tumour sections. The data are mean areas +/- SD obtained from 5 control mice and 5 mice treated with P111-136. **p < 0.01 versus control.

**Figure 4:**
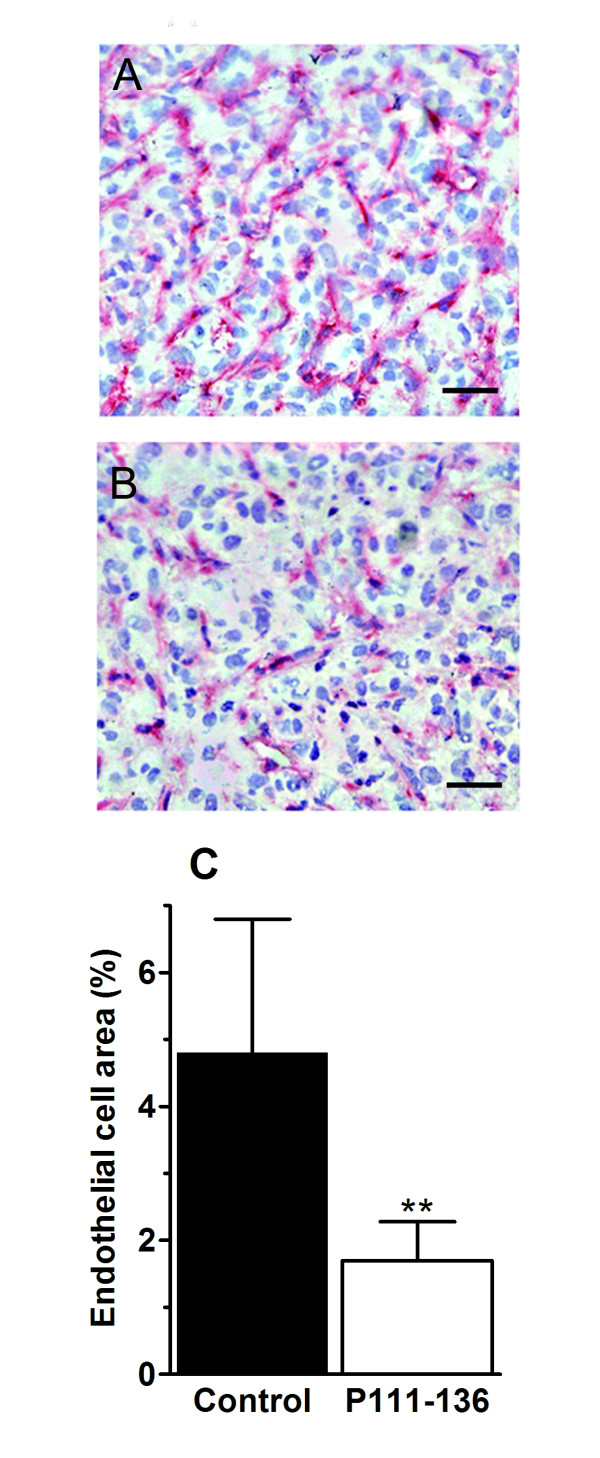
**Inhibition of tumour angiogenesis by peptide P111-136**. For immunohistochemical analysis, tumour sections were prepared and stained with anti-CD31 antibodies. (A), untreated tumours and (B), tumours treated with P111-136. Scale bar, 100 μm. (C), angiogenesis was quantified by image analysis of CD31-labelled endothelial cells. The data are mean areas +/- SD obtained from 5 control mice and 5 mice treated with P111-136. **p < 0.01 versus control.

To further investigate the effect of P111-136 on HARP-induced PC-3 proliferation *in vivo*, athymic mice were subcutaneously injected with PC-3 cells, which consistently led to tumour development within 2 weeks. P111-136 treatment was initiated at the end of the second week, when the tumours were well established, in order to simulate a curative treatment. Peritumoral injection of P111-136 (5 mg/kg/day) significantly reduced tumour growth as soon as the first week of treatment, compared to PBS used as the control (Figure 2A). After 25 days, tumour size was reduced by 61% in the P111-136 group. P111-136 treatment had no effect on body weight (data not shown) and induced no evidence of toxicity such as diarrhoea, infection, weakness, or lethargy. As expected, control treatment with Taxol^® ^(10 mg/kg twice a week) strongly inhibited tumour growth, by 71% compared to PBS, after 25 days of treatment (Figure 2A). At the end of the study, the animals were sacrificed and tumour weight was determined. Both P111-136 and Taxol^® ^significantly decreased tumour weight, by more than 65%, compared with PBS, supporting the tumour-size data (Figure 2B). In order to investigate whether P111-136 treatment was associated with apoptosis of PC-3 cells, cleaved caspase 3 immunostaining was performed on tumour sections and quantified by software analysis. As shown in Figure 3, a two-fold increase in cleaved caspase 3 labelling was observed in tumour treated with P111-136 compare to the untreated tumours. In third investigation, to determine whether angiogenesis associated with tumour growth was also affected by P111-136 treatment, we used CD31 immunostaining to quantify blood vessels. Compared to the untreated tumours (Figure 4A and 4C), peritumoral injections of P111-136 significantly decreased endothelial-cell density (Figure 4B and 4C). The mean percentage of endothelial cells in viable fields of tumours treated with 5 mg/kg/day of P111-136 was inhibited by 64% compared to the control tumour value (1.7 ± 0.58 vs. 4.8 ± 2, based on 25 fields in each of four tumours). These results suggested that the antitumoral effect of P111-136 could also act through direct inhibition of angiogenesis.

### The peptide P111-136 inhibits the *in vivo *HARP-induced angiogenesis in a Matrigel™ plug model

**Figure 5:**
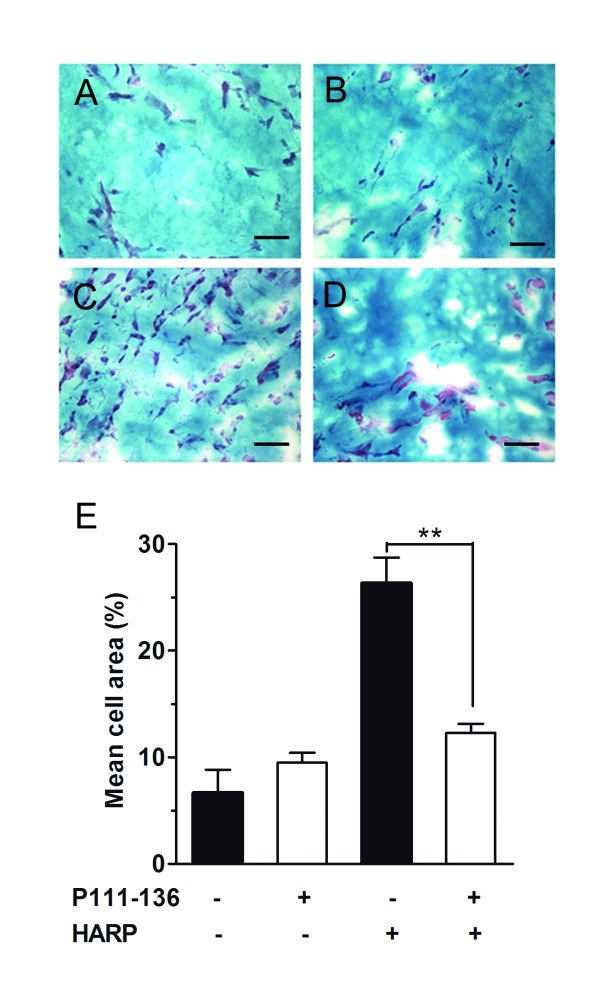
**Inhibition of HARP-induced *in vivo *angiogenesis by peptide P111-136**. Liquid Matrigel™ at 4°C was injected subcutaneously into Swiss mice. The Matrigel™ was used alone or after incorporation of HARP, P111-136, or both. The animals were sacrificed 8 days later; the Matrigel™ plugs were removed, sectioned, stained using the Gomori-Trichrome method, and examined under the microscope. Scale bar, 100 μm. Micrographs of Gomori-Trichrome stained Matrigel™ plug (A) alone, (B) containing 1 μM of P111-136, (C), containing 5 nM of HARP, or (D) containing 5 nM of HARP and 1 μM of P111-136. (E) endothelial-cell migration into the Matrigel™ was quantified as the mean cell count in six fields in each of three Matrigel™ plug sections per mouse, data are means +/- SD of values in four mice per group. **p < 0.01.

To investigate a direct effect of P111-136 on angiogenesis processes, we questioned whether the peptide inhibits the normal angiogenesis induced by HARP. This study was performed using *in vivo *mouse Matrigel™ plug assay. Incorporating 5 nM of HARP into the Matrigel™ resulted in a 3.7-fold increase in endothelial-cell infiltration (Figure 5C and 5E) compared to the untreated control plug, which contained only a few endothelial cells (Figure 5A and 5E). Addition of 1 μM of P111-136 to the HARP-containing Matrigel™ inhibited the effect of HARP on endothelial cell infiltration by 72% (Figure 5C, D and 5E). P111-136 had no effect on endothelial-cell infiltration using Matrigel™ alone (Figure 5A, B and 5E). These data indicate that P111-136 inhibited the angiogenesis induced by HARP.

### P111-136 targets HARP interacting proteins

**Figure 6:**
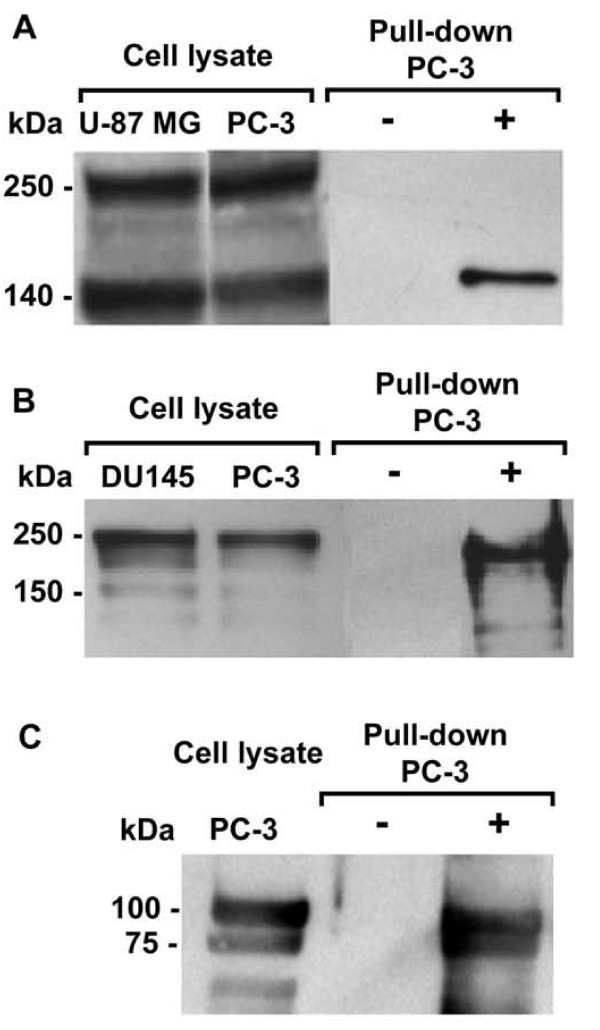
**Interaction of P111-136 with HARP receptors**. Whole PC-3 cell lysates were incubated or not with biot-P111-136 immobilized on streptavidin-Sepharose beads, or with streptavidin-Sepharose beads alone. The lysates or the precipitates were analyzed by SDS-PAGE and electroblotted and the membrane was probed against ALK (A), RPTPβ/ζ (B) and nucleolin (C) using specific antibodies. U87 MG and DU145 cell lysates were respectively used as positive controls for the presence of ALK and RPTPβ/ζ receptors.

Since P111-136 was previously described to compete with HARP for the binding of the ALK receptor [26] in a cell free assay, we then questioned whether P111-136 could bind *in situ*, using PC-3 cells, ALK and also the other molecular targets involved in the biological activity of HARP including RPTPβ/ζ and nucleolin [34]. Pull-down experiments using biotinylated P111-136 (biot-P111-136) and Western blot analysis were performed to answer this question. Firstly, Western blot analysis performed from whole-cell extracts of PC-3 indicated that this cell line expressed the 140 and 220 kDa forms of ALK (Figure 6A), only the 240 kDa form of RPTPβ/ζ (Figure 6B) and the 100 kDa nucleolin and its degradation products (Figure 6C), as previously described [35]. For expression of ALK and RPTPβ/ζ, U87 MG and DU145 cell lysates were used respectively as control (Figure 6A and 6B). These different HARP interacting proteins were also detected in biot-P111-136 pull down experiments (Figure 6A, B and 6C) in which these proteins were identified by Western blot analysis while no band was detected when bio-P111-136 was omitted from the assay. It is noteworthy that only the 140 kDa isoform of ALK was detected in the biot-P111-136 pull down (Figure 6A). All together these results demonstrate that the inhibition of PC-3 proliferation observed with P111-136 could be link to a direct or indirect binding of this peptide to the different HARP interacting proteins.

## Discussion

HARP is expressed in a wide range of tumour cell lines including neuroblastoma, glioblastoma, melanoma and also of cancers derived from the pancreas, breast, and prostate [8,10,11,36-38]. Numerous studies using ribozyme, RNA interference or antisense strategies [12,38-41] showed that HARP was a potential *in vivo *rate-limiting angiogenic factor in tumour growth and metastasis. As a result, HARP and its two signalling receptors ALK and RPTPβ/ζ are now viewed as promising targets for cancer therapy [17,38,39,42,43]. Using recombinant deletion mutant of HARP, we previously established that the HARP C-terminus domain, composed of amino acids 111 to 136, was closely involved in the mitogenic, angiogenic and transforming activities of HARP [25,26]. Thus, the synthetic peptide P111-136 has been shown to bind to the ALK receptor, thereby acting as a dominant inhibitor of the biological activities of HARP. These findings, together with the key role for HARP in prostate-tumour growth, prompted us to further investigate the potential anti-tumour effects of P111-136, using the human androgen-independent adenocarcinoma PC-3 cell line, which expressed HARP and its receptors [11].

In the *in vitro *experiments reported here, P111-136 inhibited the growth of PC-3 cells. Using whole-cell lysate from PC-3 cells, pull-down experiments with biotinylated P111-136 indicated that P111-136 interact, as expected, with ALK and also with RPTPβ/ζ suggesting that this peptide might bind also to the RPTPβ/ζ receptor. This hypothesis is strengthen by the recent data showing that synthetic peptide including amino acids 122 to 131 derived from the C-terminus region of HARP binds to RPTPβ/ζ [29]. Recently Diamantopoulou et al [33] have demonstrated that this shorter peptide P122-131 was able to inhibit the proliferation of other human prostate cancer cell lines DU145 and LNCaP. It is interesting to mention that these cell lines do not express the ALK receptor (Additional file 1, Figure S1). However, when tested in our conditions, P122-131 inhibited only the anchorage independent growth of PC-3 cells by 22% at 1 μM when compare to 47% for P111-136. The presence of the ALK receptor in PC-3 cells could explain this difference of efficiency between the two peptides but further investigations of both peptides on these different prostate cancer cell lines will be need to clarify this point. When assayed on the human glioblastoma cell line U87 MG described previously as expressing a low level of RPTPβ/ζ [44], P111-136 inhibited the colony formation in a lesser extent (data not shown) supposing that an optimal expression of both ALK and RPTPβ/ζ receptors could be important for a strong effect of P111-136. Therefore, further investigations will be needed to verify this assumption. For several years, the receptors involved in the growth-promoting effect of HARP are a matter of controversy [45] due to the cell system and molecular tools used. However, it has been clearly demonstrated that the binding of HARP or its related protein MK to the ALK receptor activated the intracellular kinase domain and further stimulated the downstream MAP and PI-3 kinase pathways [13,46,47]. More, recently Stylianou et al., have also mapped the HARP ligand binding domain on ALK and found that a single chain antibody was able to compete for the HARP binding and inhibit its intercellular downstream signal [48]. Other data have reported that binding of HARP to RPTPβ/ζ receptor oligomerised the receptor and inactivated its intracellular catalytic phosphatase activity, leading to further activation of the Src/Fyn kinase family and β-catenin phosphorylation pathway [20,21]. The RPTPβ/ζ receptor was found to be involved in HARP-induced cell migration and neurite outgrowth [18,19] and was then shown to play a role in glioblastoma cell proliferation [42,43]. In addition, a recent study [24,42,43] identified an alternative mechanism in which binding of HARP to RPTPβ/ζ maintained the phosphorylation of ALK, leading to further activation of its pathway. The results described in this study, strongly suggested that a link between ALK and RPTPβ/ζ could occur in PC-3 cells since P111-136 pulled down both receptors.

Nucleolin, a nuclear and cytoplasmic protein initially related to rRNA maturation ribosome assembly [49], was also described as a cell surface protein [35] and as a low affinity binding protein for HARP [34] and MK [50,51]. Since nucleolin was clearly identified in our pull down experiments, it is tempting to suggest that this protein could also be implicated in the HARP biological activities. As support of this hypothesis, we have shown that anti nucleolin antibody inhibited the biological activity of HARP in MDA-MB 231 cell line [52]. The mechanism of this inhibition remains to be studied. However, previous data have shown that the β-sheet domains of HARP, especially those located on the C-terminal part of the molecule, were involved in the binding of HARP to nucleolin. Since nucleolin was pulled down by P111-136 in our experiments, we could speculate that the peptide did not bind directly to nucleolin but through other non identified molecules, this possibility is currently under evaluation.

The strong inhibition of P111-136 observed on the growth of PC-3 cells *in vitro *and their tumour xenograft in nude mice could be explain because it targets directly or indirectly different proteins of the HARP pathway. Furthermore *in vivo*, the inhibition of tumour growth was linked with an increase in PC-3 cells apoptosis and a decreased in the tumour associated angiogenesis. During this treatment, no side effects were observed on mice suggesting that P111-136 did not present any toxicity nor cause additional pathology on animals. Recently Grzelinski et al., [53] have demonstrated an enhanced antitumor effect on glioblastoma using a double ribozyme strategy targeting HARP and one of its receptor. It is tempting to speculate that such results could be obtained on PC-3 tumours in combining P111-136 treatment with another molecule that target the HARP pathway.

Evidence that C-terminus maturation influences the biological effects of HARP was obtained in an earlier study, in which a 14-kDa C-terminal truncated form of HARP influenced the proliferation of cells like bovine epithelial lens cells [54]. Thus, natural processing of the HARP molecule could be pivotal in regulating the biodistribution and biological effects of HARP in health and disease. In keeping with this possibility, we showed that plasmin and MMP-2 cleaved HARP *in vitro*, releasing various peptides that may differentially affect the angiogenic and mitogenic activities of HARP [55,56]. GAGs in the microenvironment may protect HARP from this enzymatic cleaving. Thus, the biological effect of HARP is the net result not only of HARP secretion and degradation, but also of specific enzymatic processing, which depends on the proteases and GAGs present in the microenvironment and may generate peptides that have diverse (and perhaps opposite) biological effects.

## Conclusions

Our results demonstrate that P111-136 strongly inhibits the proliferative effect of HARP on *in vitro *and *in vivo *growth of PC-3 cells. This inhibition of P111-136 could be link to a direct or indirect binding of this peptide to the different HARP interacting proteins (ALK, RPTPβ/ζ, nucleolin). *In vivo*, the daily P111-136 treatment was as effective as the one of a clinical drug used in prostate cancer therapy. Furthermore the tumour growth inhibition was associated with an inhibition of angiogenesis and an increase in PC-3 cells apoptosis. Thus, P111-136 may be considered as an interesting pharmacological tool to interfere with tumour growth that has now to be evaluated in other cancer types and animals models.

## List of abbreviations

HARP: heparin affin regulatory peptide; DMEM: Dulbecco's modified Eagle medium; PBS: phosphate buffer saline; ALK: anaplastic lymphoma kinase; RPTPβ/ζ (Receptor Protein Tyrosine Phosphatase β/ζ);

## Competing interests

The authors declare that they have no competing interests.

## Authors' contributions

YHK was the major contributor of this work in designing, executing experiments, interpreting the results and contributing to drafting the manuscript. OB contributed to experiments and participated in interpretation of the results. IBP contributed to the development of this study and to the first experiments. RK and SF provided assistance to YHK and OB for the experiments. DRM contributed to analysis, interpretation of the results and was involved in revising the manuscript. JC and JD were responsible for this study, participating in the design, the analysis, and the interpretation of the results, drafting and overseeing all stages of the revision of the manuscript. All authors read and approved the final manuscript.

## Pre-publication history

The pre-publication history for this paper can be accessed here:

http://www.biomedcentral.com/1471-2407/11/212/prepub

## Supplementary Material

Additional file 1 **Expression of the ALK receptor in PC-3, DU145 and LNCap cells**. DU145 and LNCaP were cultured in completed medium as described in Diamantopoulou et al., [33]. Western blot (WB) and RT-PCR experiments were performed with respectively lysate and total RNA from PC-3 (positive control), DU145 and LNCaP cells as described in Dos Santos et al., [28]. Click here for file
