# Supplementary material for: The synthetic peptide P111-136 derived from the C-terminal domain of heparin affin regulatory peptide inhibits tumour growth of prostate cancer PC-3 cells
Source: BMC Cancer. 2011 May 30;11:212. doi: 10.1186/1471-2407-11-212 (PMC3118947; doi:10.1186/1471-2407-11-212)
Supplement: Additional file 1 — Expression of the ALK receptor in PC-3, DU145 and LNCap cells. DU145 and LNCaP were cultured in completed medium as described in Diamantopoulou et al., [33]. Western blot (WB) and RT-PCR experiments were performed with respectively lysate and total RNA from PC-3 (positive control), DU145 and LNCaP cells as described in Dos Santos et al., [28]. [file 1471-2407-11-212-S1.PDF]

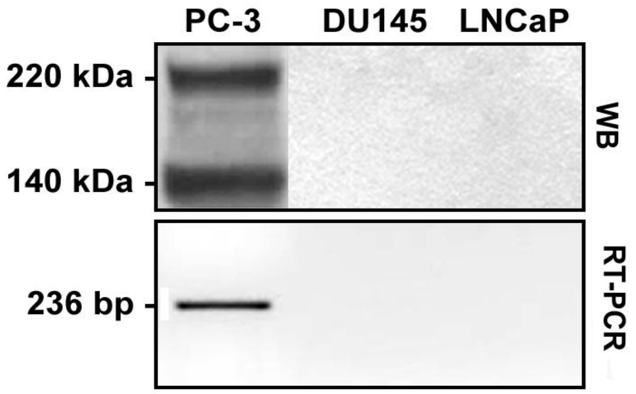


**Figure S1. Expression of the ALK receptor in PC-3, DU145 and LNCap cells.** DU145 and LNCaP were cultured in completed medium as described in Diamantopoulou et al., [33]. Western blot (WB) and RT-PCR experiments were performed with respectively lysate and total RNA from PC-3 (positive control), DU145 and LNCaP cells as described in Dos Santos et al., [28].
